# Supplementary material for: Mapping gene expression across the microbiome–gut–brain axis of germ-free and specific pathogen-free mice
Source: Microbiology (Reading). 2026 Sep 29;172(9):001778. doi: 10.1099/mic.0.001778 (PMC13623194; doi:10.1099/mic.0.001778)
Supplement: Supplementary file 1 [file mic-172-01778-s001.pdf]

Mapping gene expression across the microbiome-gut-brain axis of germ- and specific pathogen-free mice

Clio Dritsa[1], Scott Hoffmann[2], Heather Hulme[2], Connor Lynch[3], Vicky Taylor[3], Richard Burchmore[1], John Cole[1], Richard J.A. Goodwin[2], Daniel M. Wall[1,*]

[1]School of Infection and Immunity, College of Medical, Veterinary and Life Sciences, University of Glasgow, United Kingdom
[2]Imaging and Data Analytics, Clinical Pharmacology and Safety Sciences, R&D, AstraZeneca, Cambridge, United Kingdom
[3]Biological Services Facility, The University of Manchester, Manchester, United Kingdom.

Supplementary Materials

Supplementary Figure 1

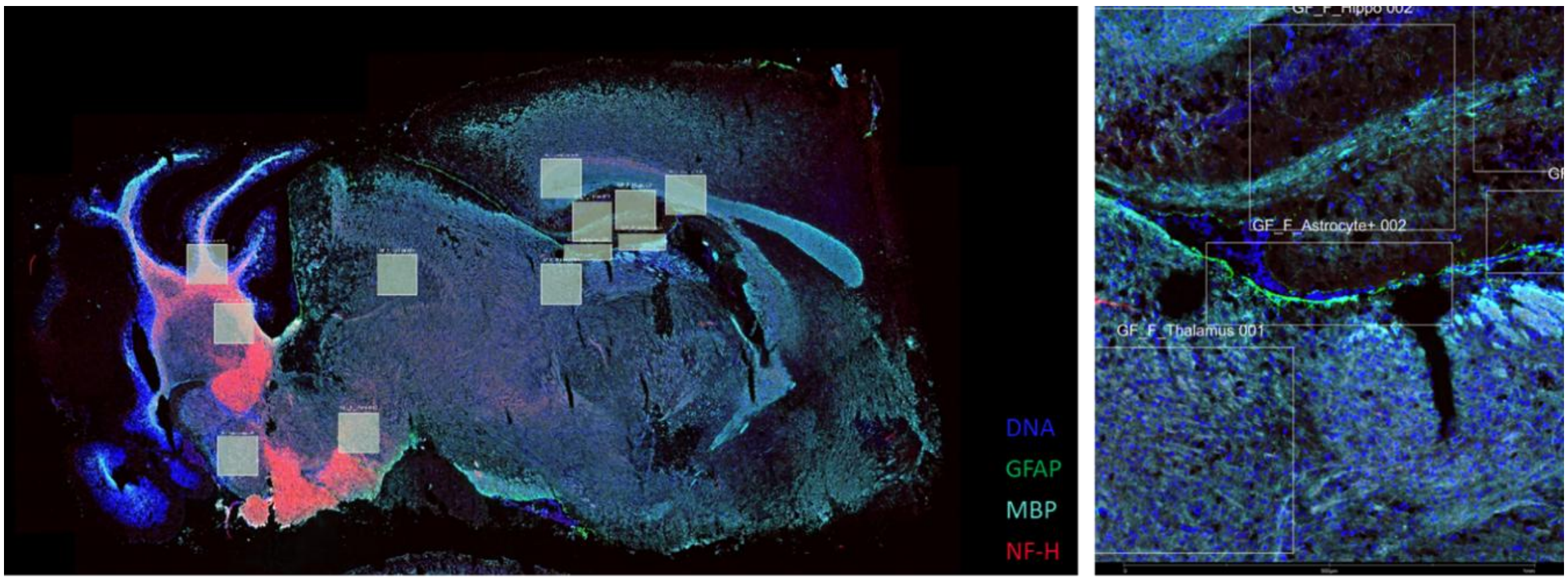

Supplementary Figure 1. Multiplex Immunofluorescence (mIF) image taken on the Digital Spatial Profiler (DSP) platform. Representative image of a 10 μM sagittal section of adult mouse brain using GFAP (green), MBP (light blue), NFH (red) and nucleic acid immunofluorescence marker (dark blue) captured on the Nanostring DSP for morphological identification of structures. The mask square boxes indicate ROIs that were profiled on the Nanostring GeoMx platform using the GeoMx Mouse Whole Transcriptome Atlas.

Supplementary Figure 2

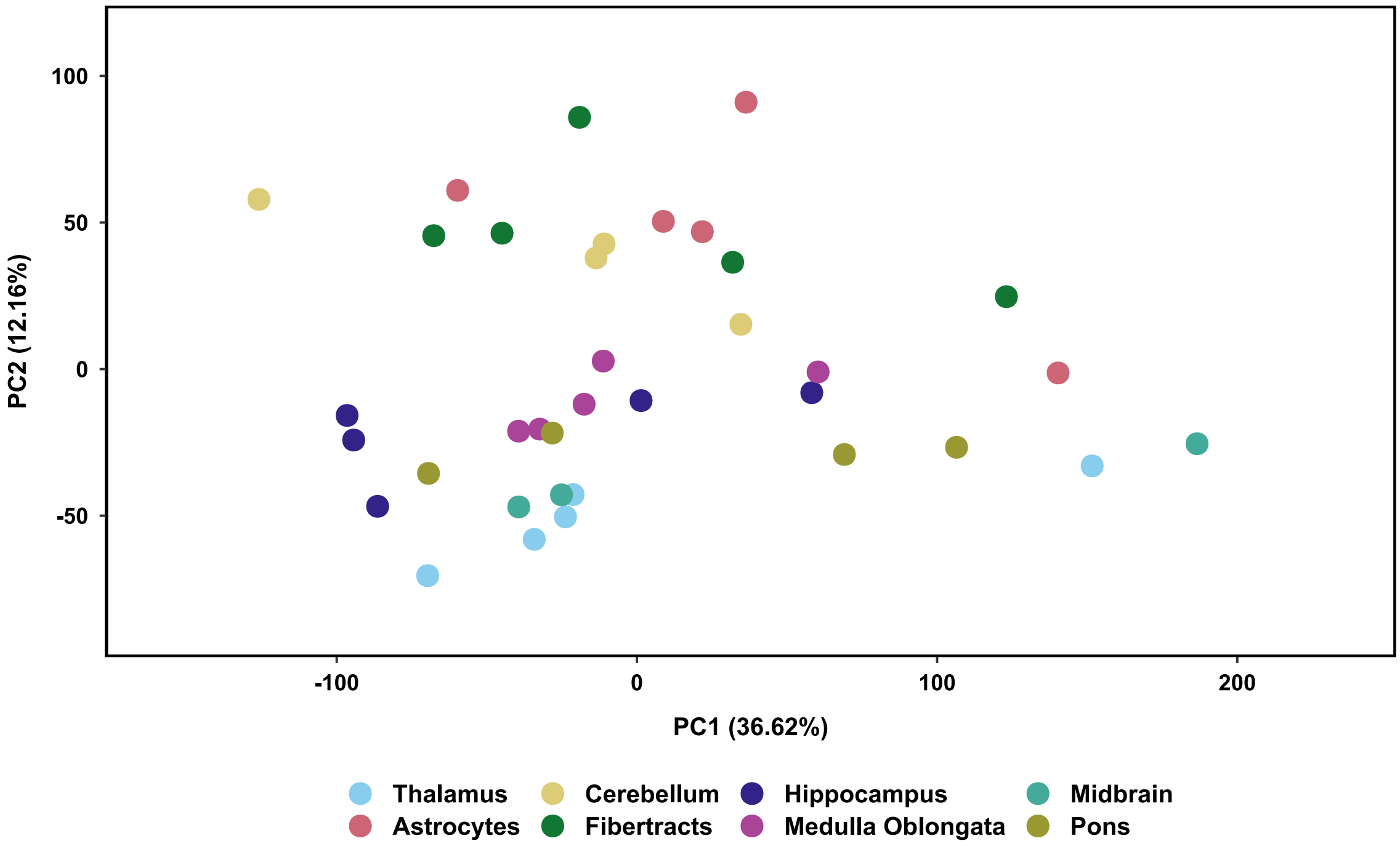

Supplementary Figure 2. Assessing variation within the data by PCA plot. PCA of gene expression in specific brain regions of a representative SPF mouse brain.

Supplementary Figure 3

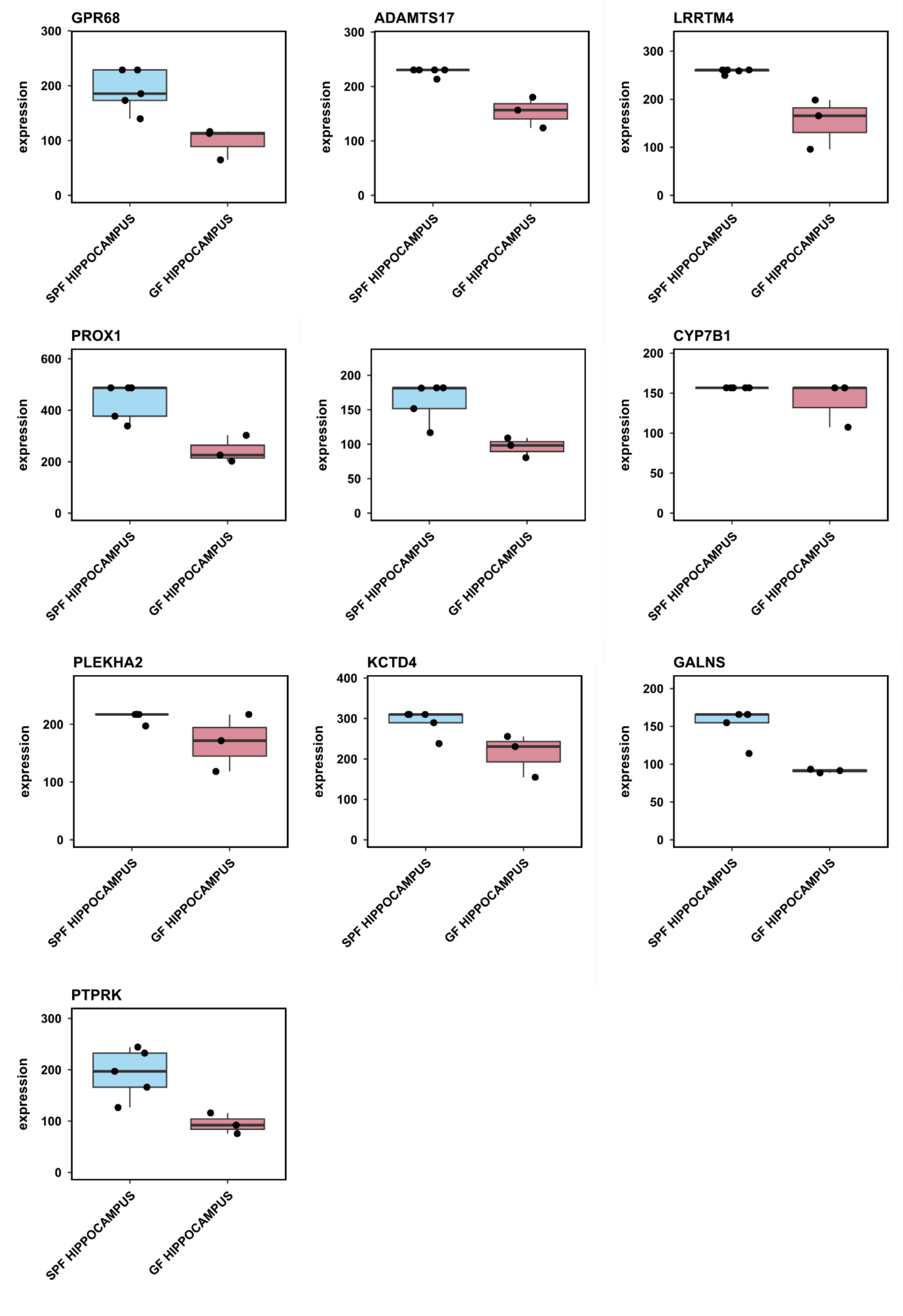

Supplementary Figure 3. Boxplots of top ten DEGs with significantly lower expression in the GF compared to the SPF hippocampus. Boxplots showing GF hippocampus n=3 (red) and SPF hippocampus n=5 (blue) for the ten most significantly decreased DEGs in GF hippocampus (*p*. adj < 0.05, absolute log2fold > 0.5). Each dot is one sample, with sample groups shown on x-axis and gene expression values on the y-axis with mean (parallel line) and standard error (vertical to x-axis) for each sample group.

Supplementary Figure 4

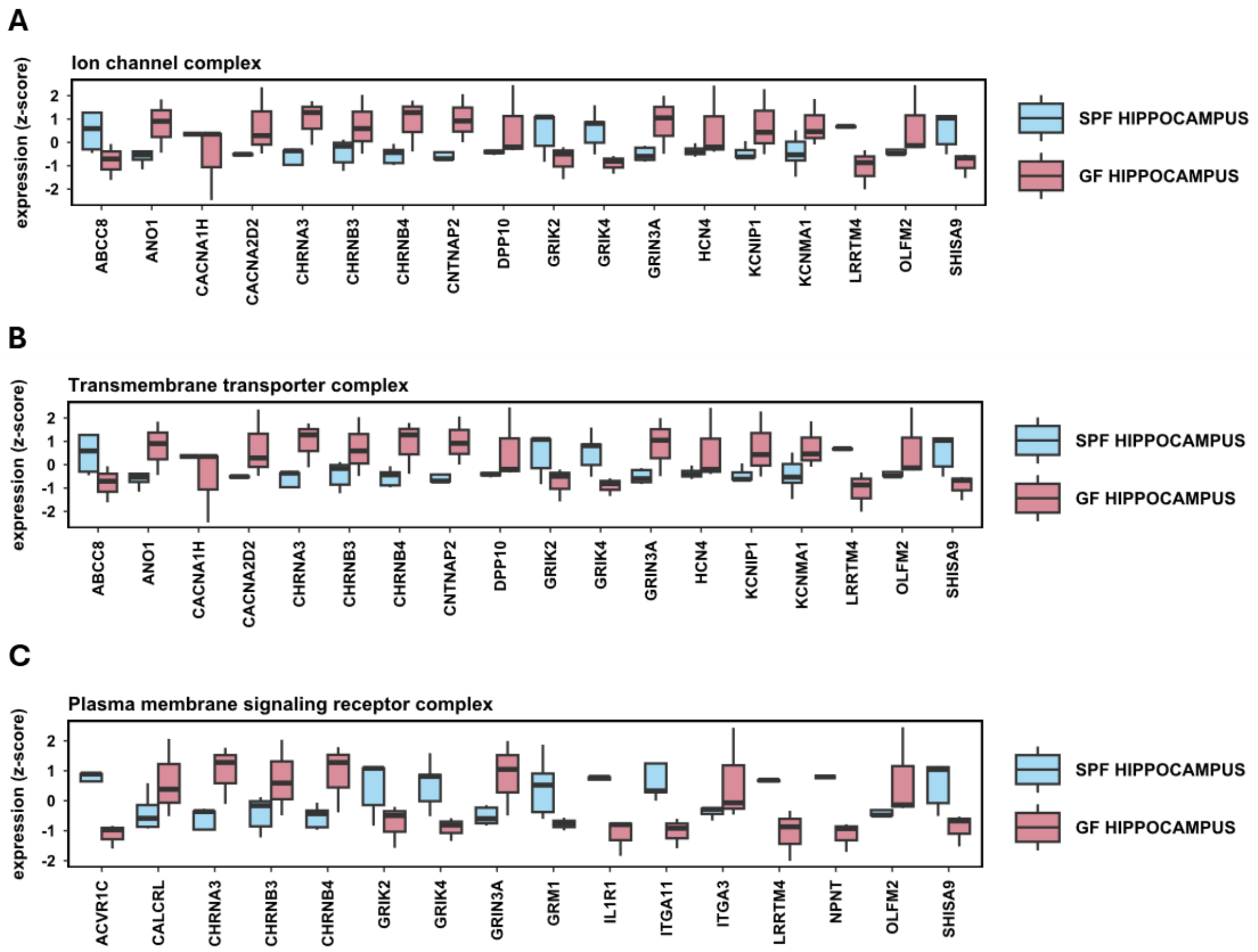

Supplementary Figure 4. ORA summary boxplots of DEGs for the top three most enriched gene sets for the comparison of GF and SPF hippocampus. Representative summary boxplots with expression values in z-scores to illustrate the three top, most enriched genes sets for all significantly DEGs (*p.* adj 0.05, absolute log2 fold > 0.5) between GF hippocampus and SPF hippocampus, significantly upregulated genes and significantly downregulated genes. (**A**) Ion channel complex gene set. (**B**) Transmembrane transporter complex gene set. (**C**) Plasma membrane signalling receptor complex gene set. For each plot the significantly differential genes within each gene set are provided on the x-axis and expression values scaled into per gen z-scores are shown on the y-axis. Boxes are shown for GF hippocampus (red) and SPF hippocampus (blue) groups. Enrichment analysis was performed using Hypergeometric Gene Set Enrichment on the gene set databases STRING11.5. The figure provides a visual representation of the DEGs comprising 3 out of 5 gene sets taken from Figure 3B, namely the ion channel complex, the transmembrane transporter complex and the plasma membrane signalling receptor

complex. It was observed that the DEGs within these gene-sets were primarily highly expressed in the GF hippocampus versus SPF hippocampus. The DEGs between ion channel complexes and transmembrane transporter complexes gene sets were shared (seen in Figure 8A and Figure 8B). The plasma membrane signalling receptor gene set (Figure 8 C), comprised many of the DEGs seen in the first and second most enriched categories (Figure 8A and Figure 8B) such as *Chra3*, *Chrnb3*, *Chrnb4*, *Grik2*, *Grik4*, *Grin3a*, *Llrtm4*, *Olfm2* and *Shisa9*. However, the plasma membrane signalling receptor gene set also comprised DEGs that were not seen in the first two enriched categories, namely *Acvr1c*, *Calcrl*, *Grm1*, *IL1R1*, *Itga11*, *Itga3* and *Npnt*. While individual DEGs showed higher log fold changes (Table 2), ORA analysis indicated that DEGs within functional categories of genes sets were significantly changing, but at lower levels (Figure 8).

Supplementary Figure 5

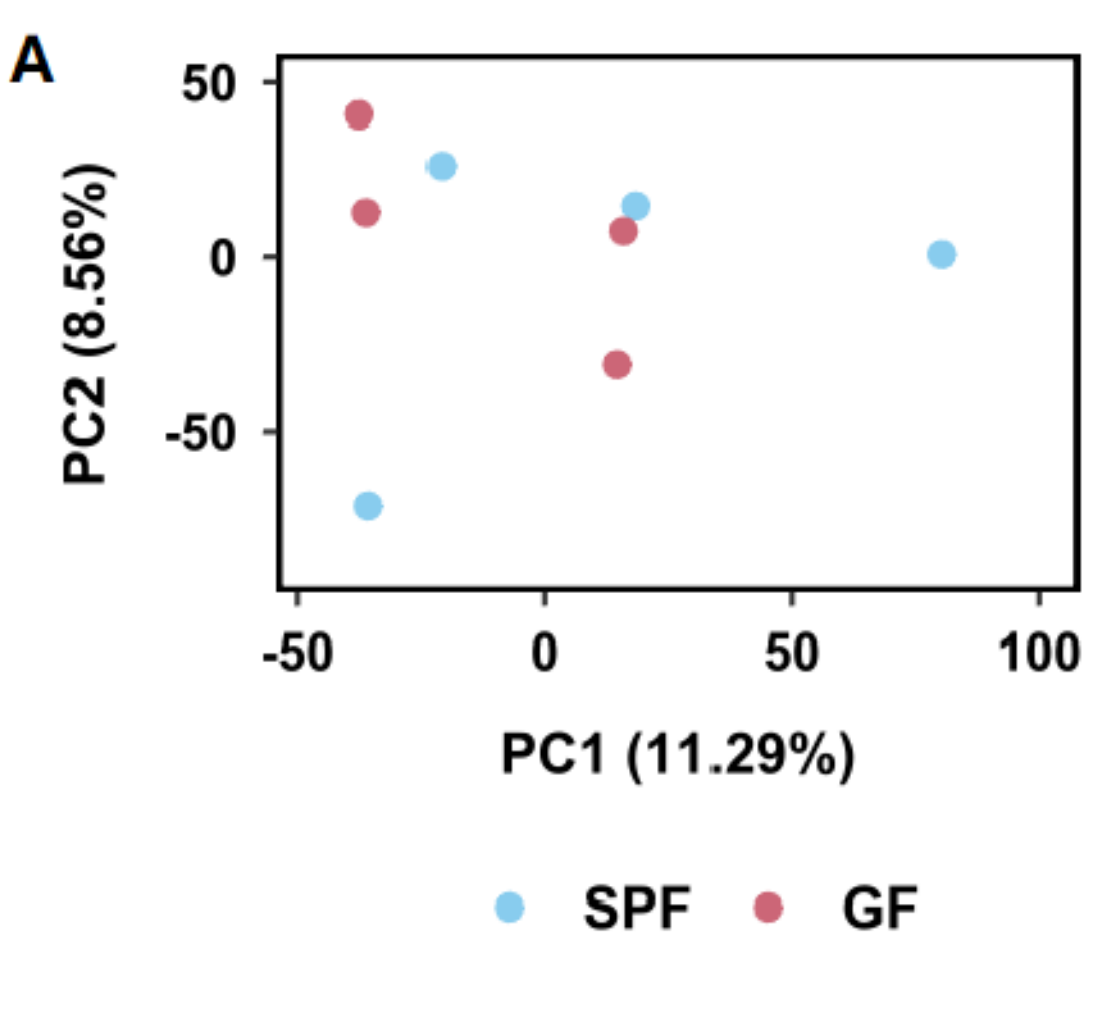

Supplementary Figure 5. Clustering of DEGs from comparison of the GF and SPF pons. PCA plot of GF pons n=4 (red) and SPF pons n=4 (blue), showing the first principal component (PC1) accounting for 50.44 % of variance and the second principal component (PC2) capturing 20.87% of variance.

Supplementary Figure 6

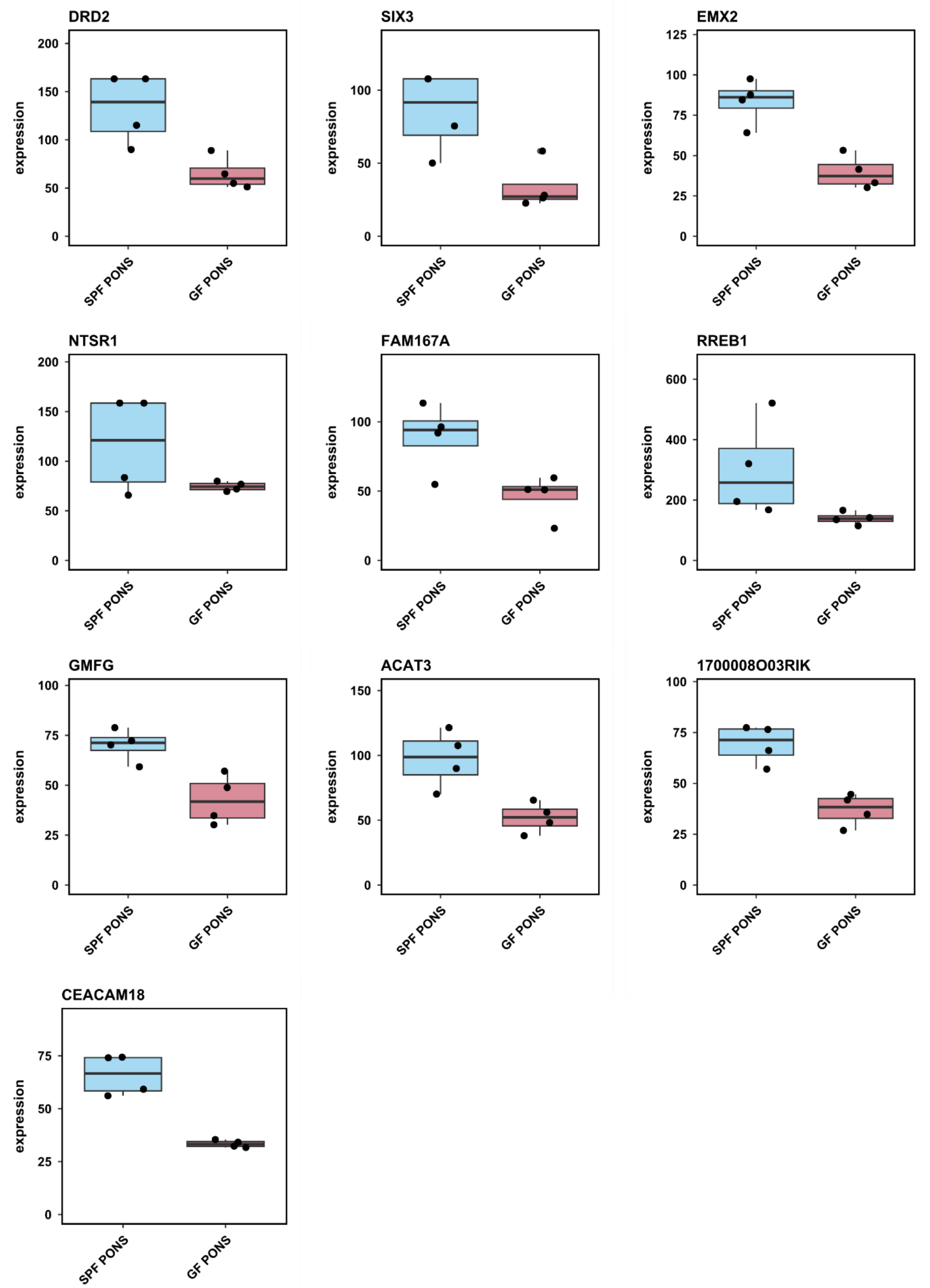

Supplementary Figure 6. Boxplots of top ten DEGs with significantly lower expression in the GF compared to the SPF pons. Boxplots of gene expression values of GF pons n=4 (red) and SPF pons n=4 (blue) for the ten most highly expressed DEGs found in GF pons, (*p*. adj < 0.05, absolute log2fold > 0.5). Each dot is one sample, with sample groups shown on x-axis and gene expression values on the y-axis with mean (parallel line) and standard error (vertical to x-axis) for each sample group.

Supplementary Figure 7

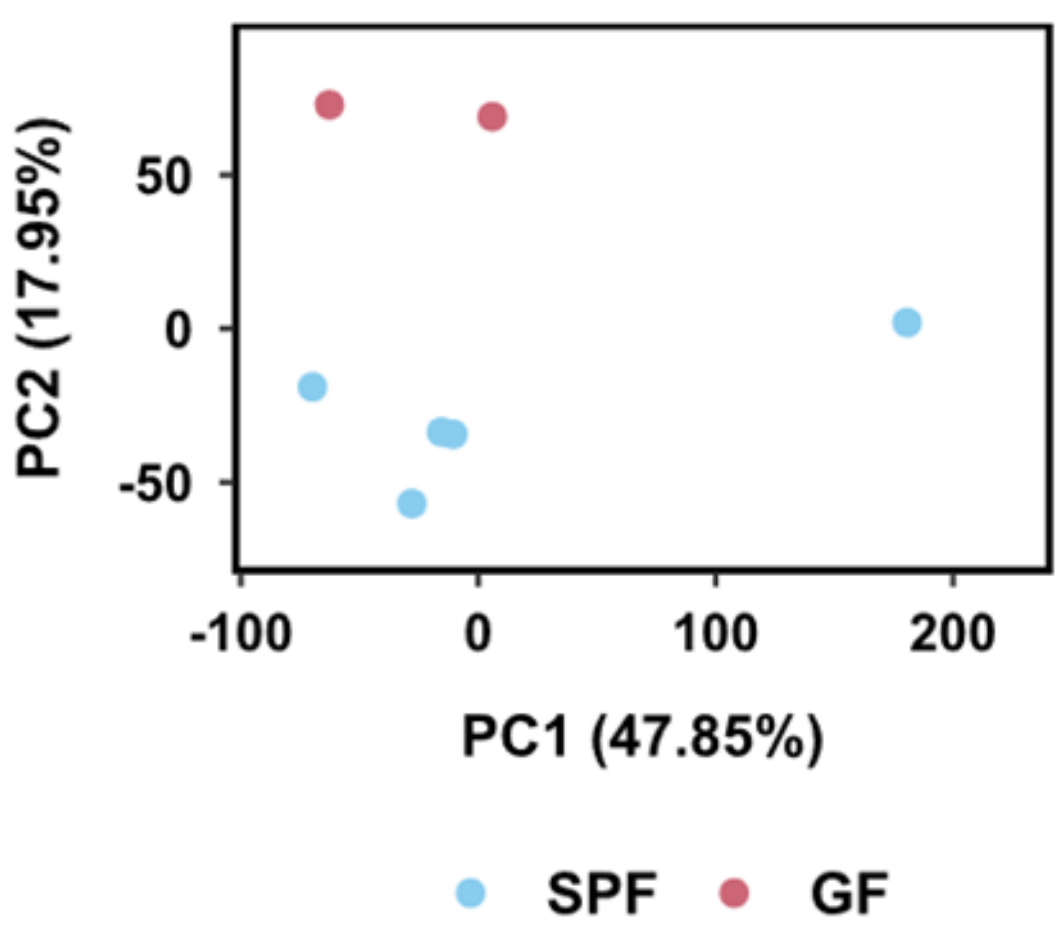

Supplementary Figure 7. Sample clustering of DEGs between GF and SPF thalamus. PCA plot of GF n=2 (red) and SPF n=5 (blue) thalamus samples, showing the first principal component (PC1) accounting for 47.85% of variance and the second principal component (PC2) capturing 17.95% of variance.

Supplementary Figure 8

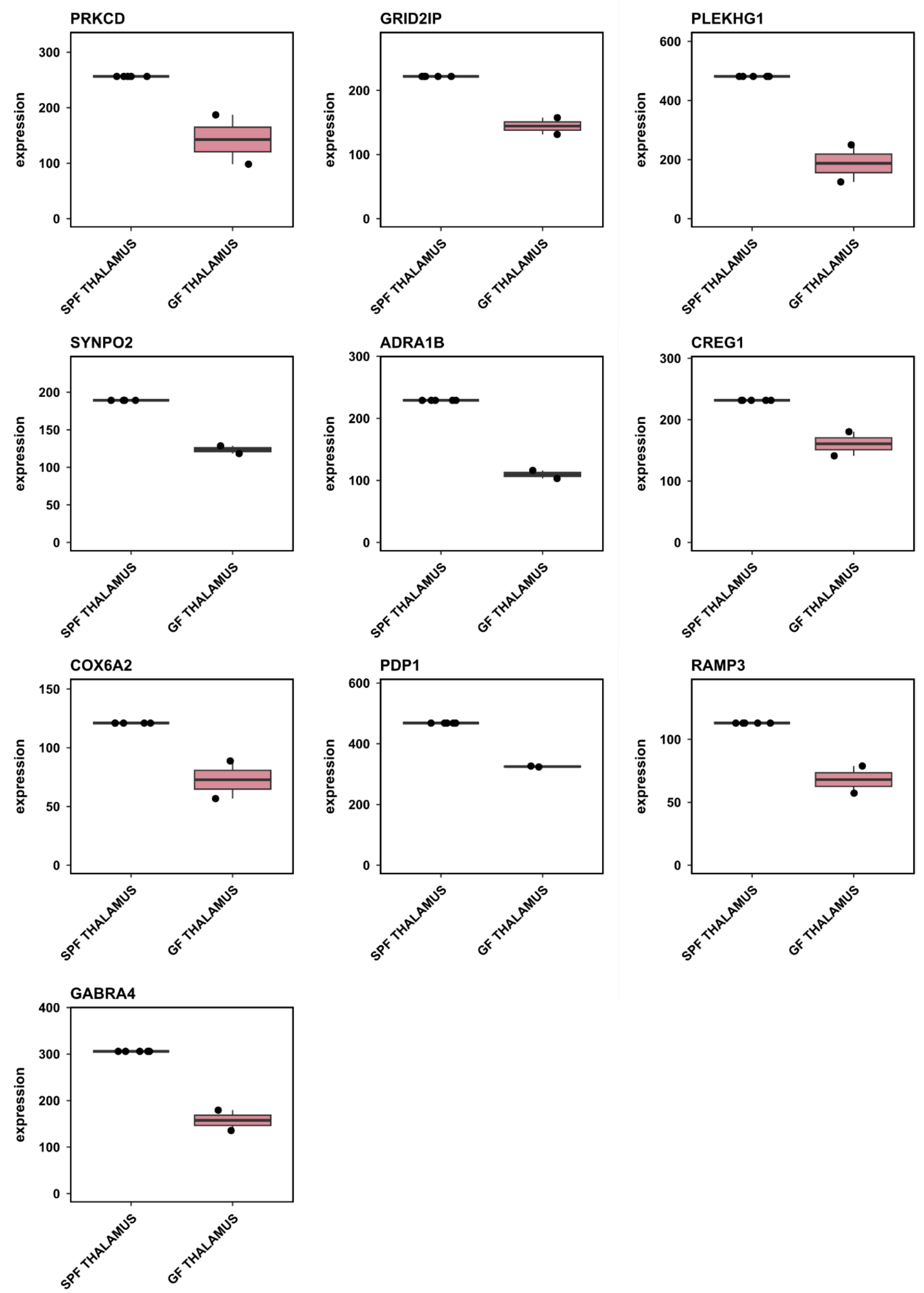

Supplementary Figure 8. Boxplots of top ten DEGs with significantly lower expression in the GF compared to the SPF thalamus. Boxplots of gene expression value of GF thalamus n=2 (red) and SPF thalamus n=5 (blue) for the ten most highly expressed genes found in GF thalamus by adjusted *p*-value, ($p < 0.05$). Each dot is one sample, with sample groups shown on x-axis and gene expression values on the y-axis with mean (parallel line) and standard error (vertical to x-axis) for each sample group.

Supplementary Table 1

	Mice		ROIs	
	**GF**	**SPF**	**GF**	**SPF**
Hippocampus	3	5	7	17
Thalamus	2	5	2	7
Pons	4	4	5	6

Supplementary Table 1. Summary table of mouse numbers for each brain region and number of ROIs selected across these samples. In total 4 GF and 5 SPF mice were used in this study but for all mice it was not possible to identify ROIs due to sectioning artefacts. For example, 17 ROIs were selected for the SPF hippocampus across the 5 SPF mice.

Supplementary Table 2

Gene	Function Summary
**SYT9**	Synaptotagmin family calcium-binding synaptic vesicle protein. A calcium sensor for classical neurotransmitter release, it localizes to dense-core vesicles and regulates calcium-triggered release of neuropeptides/hormones.
**CHRNA3**	An alpha-subunit that co-assembles with CHRNB4 and CHRNB2 to form pentameric, ligand-gated nicotinic acetylcholine receptors with high calcium permeability. These channels mediate fast excitatory cholinergic transmission, influence dopamine signalling and appetite regulation, and are linked to nicotine dependence.
**SYT6**	A calcium- and phospholipid-binding member of the synaptotagmin family with a transmembrane domain and tandem C2 domains. Again implicated in calcium-regulated vesicle trafficking/exocytosis.
**PRKCQ**	A serine/threonine kinase of the novel PKC family which is activated by diacylglycerol.
**NRP1**	A transmembrane co-receptor that partners with plexins or VEGF receptors to regulate axon guidance, cell migration, and angiogenesis.

**CHRNB4**	A beta-subunit that assembles with alpha subunits (especially CHRNA3 described above) to build heteropentameric nicotinic acetylcholine receptors mediating fast synaptic.
**MINAR1**	A largely intrinsically disordered, single-pass transmembrane cell-surface receptor that binds and stabilizes NOTCH2, thereby modulating Notch signalling.
**KCNIP1**	A cytosolic, calcium-binding neuronal calcium sensor protein that associates with potassium channel subunits to regulate their trafficking, density, and inactivation/recovery. Acts in a calcium-dependent manner.
**IRX1**	A homeodomain-containing transcription factor of the Iroquois family that has primarily been implicated in regulation of patterning and differentiation during embryonic development.
**TAC2**	Encodes a preprotachykinin that is proteolytically processed to release neurokinin B, a tachykinin-family neuropeptide. Involved in neurotransmission linked to stress responses, fear memory, and social/aggressive behaviour.

Supplementary Table 2: Supplementary Table providing extended detail for the ascribed function of expressed genes that were identified in Table 2; DEGs most significantly changed in the GF compared to the SPF hippocampus.

Supplementary Table 3

Gene	log2fold	p	p.adj
GPR68	-1.11	8.016103e-09	7.730607e-06
ADAMTS17	-1.17	1.376545e-08	1.232696e-05
LRRTM4	-1.58	6.794341e-08	4.696261e-05
PROX1	-1.43	5.836582e-07	2.090664e-04
MKX	-1.13	1.082724e-06	3.572135e-04
CYP7B1	-0.97	2.268044e-06	6.612668e-04
PLEKHA2	-1.10	2.427025e-06	6.915366e-04
KCTD4	-1.21	2.643806e-06	7.208062e-04
GALNS	-1.06	2.778258e-06	7.298252e-04
PTPRK	-1.02	3.610638e-06	8.230285e-04

Supplementary Table 3. Top ten most significantly decreased DEGs in the GF compared to SPF hippocampus. Ten most significantly decreased DEGs with cut off *p*. adj <0.05, absolute log2 fold > 0.5.

Supplementary Table 4

Gene	Function Summary
**NXPH4**	A secreted, brain-enriched neuropeptide-like glycoprotein that modulates cerebellar inhibitory (GABAergic) synapses, contributing to motor learning and coordination.
**MEIS2**	A TALE-class homeodomain transcription factor that partners with PBX and HOX proteins to stabilize homeoprotein–DNA complexes, driving transcriptional programs in development and differentiation, including in the brain.
**LAMP5**	A lysosome/endosome-associated membrane glycoprotein enriched in neurons, where it localizes to synaptic vesicles in a subset of GABAergic neurons and is implicated in vesicle trafficking and organelle localization.
**GAL**	A neuropeptide precursor processed into galanin and galanin message-associated peptide (GMAP). Galanin acts on three G protein-coupled receptors (GALR1–3) to inhibit neurotransmitter release, modulating feeding, sleep/wake state, nociception, mood, and neuroendocrine hormone secretion.
**CALCR**	A G protein-coupled receptor activated by calcitonin. In the brain it contributes to non-aversive suppression of food intake and energy balance.
**GPX3**	A secreted selenoprotein and the only extracellular member of the glutathione peroxidase family. It protects tissues from oxidative damage.
**CYB561**	A six-transmembrane, di-heme electron-transport protein that uses ascorbate as an electron donor to regenerate monodehydroascorbate and/or reduce ferric iron transport across membranes. Supports neuroendocrine secretory vesicle function and iron metabolism.
**GABRQ**	A subunit of the heteropentameric GABA-A receptor, the ligand-gated chloride channel mediating fast inhibitory neurotransmission.

**SST**	A cyclic neuropeptide hormone acting through five somatostatin receptors (SSTR1–5) to broadly inhibit hormone and neurotransmitter secretion. It modulates cognition, pain, and neuroendocrine signalling.
**RLN3**	An insulin/relaxin-superfamily neuropeptide, processed into a disulfide-linked A/B chain heterodimer, expressed mainly by neurons of the brainstem nucleus incertus. Acting via its receptor RXFP3, it modulates stress responses, arousal, feeding/appetite, and memory.

Supplementary Table 4: Supplementary Table providing extended detail for the ascribed function of expressed genes that were identified in Table 3; DEGs most significantly increased in expression between the GF and SPF pons.

Supplementary Table 5

Gene	log2fold	p	p.adj
DRD2	-2.12	2.724298e-07	0.001446376
SIX3	-1.93	3.042012e-07	0.001446376
EMX2	-1.11	5.522510e-06	0.006059468
NTSR1	-1.68	3.254425e-05	0.029013201
FAM167A	-1.11	1.139700e-04	0.067736182
RREB1	-1.11	1.314015e-04	0.074972447
GMFG	-0.77	1.444158e-04	0.079228731
ACAT3	-0.92	1.684701e-04	0.086766276
1700008O03RIK	-0.94	2.261130e-04	0.099727515
CEACAM18	-1.01	2.955473e-04	1.000000000

Supplementary Table 5. Top 10 DEGs significantly decreased in expression in the GF compared to SPF pons. Ten most significant DEGs with cut off *p*. adj <0.05, absolute log2 fold >0.5.

Supplementary Table 6

Gene	Function Summary
**UBAP1L**	Predicted to bind ubiquitin and participate in ubiquitin-dependent protein degradation, but poorly characterised.

**TRAF3**	A cytoplasmic E3 ubiquitin ligase and signalling adaptor that couples TNF-receptor superfamily members and innate immune receptors to NF-κB, MAPK, and interferon regulatory factor (IRF) pathways. Mainly thought to be involved in the immune response.
**PDCD7**	A component of the minor spliceosome (U11/U12 snRNP) that also appears to be selectively regulated during glucocorticoid- and staurosporine-induced apoptosis in T-cells.
**Gm4988**	No experimentally characterized protein function currently described in UniProt or the literature; it remains uncharacterized.
**FRMPD3**	A scaffold protein that in neurons interacts with GRIP and the AMPA receptor subunit GluA2. It has been linked to susceptibility to epilepsy.
**LMO3**	A LIM-domain transcriptional cofactor of the rhombotin family. Implicated as an oncogenic driver in neuroblastoma and other cancers.
**MDGA1**	A GPI-anchored cell-adhesion glycoprotein of the developing nervous system with immunoglobulin-like, fibronectin type III, and MAM domains. It supports radial migration of cortical neurons and regulates inhibitory synapse formation by interacting with neuroligin-2, restraining excessive GABAergic synapse development.
**GPR26**	An orphan class A GPCR showing high constitutive activity, coupling to Gs and elevating intracellular cAMP; broadly expressed across brain regions, with proposed roles in neuronal signalling and possible relevance to neurodegeneration.
**ELFN1**	A postsynaptic adhesion molecule expressed by specific interneuron populations that trans-synaptically recruits presynaptic group III metabotropic glutamate receptors (mGluR7), thereby setting target-specific short-term synaptic plasticity, and also inhibits protein phosphatase 1 (PP1).
**VSTM2L**	A secreted/membrane-associated protein highly expressed in cerebral cortex and pituitary. It acts as an antagonist of the neuroprotective peptide humanin.

Supplementary Table 6: Supplementary Table providing extended detail for the ascribed function of expressed genes that were identified in Table 4; DEGs

significantly increased in expression in the GF thalamus compared to the SPF thalamus.

Supplementary Table 7

Gene	log2fold	p	p.adj
PRKCD	-4.06	2.895966e-21	3.380751e-17
GRID2IP	-3.04	7.717696e-20	4.504819e-16
PLEKHG1	-2.94	7.536185e-19	2.932581e-15
SYNPO2	-3.01	3.020681e-16	8.815859e-13
ADRA1B	-2.43	4.523080e-16	1.056049e-12
CREG1	-1.76	3.371579e-15	6.559969e-12
COX6A2	-3.00	1.079044e-14	1.799538e-11
PDP1	-2.32	2.492843e-14	3.637681e-11
RAMP3	-2.36	3.216601e-14	4.172289e-11
GABRA4	-2.64	8.405185e-14	9.812213e-11

Supplementary Table 7. Top 10 DEGs significantly decreased in expression in the GF compared to SPF thalamus. Significant DEGs with cut off *p*. adj <0.05, absolute log2 fold >0.5.
